# 

**Published:** 1913-11-01

**Authors:** 


### CONTENTS.

| | PAGE |
|---|---|
| **The Control of Private Hospitals** ... ... | 103 |
| **Hospital Statistics** ... ... ... ... ... | 104 |
| **Hospital and Institutional News,** including— ... | 105 |
| The Future Management of York County Hospital—The Status of the Workhouse Chaplain—The St. James's Railway Disaster and the Hospitals—Salaries in London County Mental Hospitals—The Crisis at Camberwell Infirmary. | |
| **Hospital Statistics** ... ... ... ... ... | 111 |
| A Suggested Remedy for the Present Chaos. | |
| **Why an Orthopædic Department is Necessary** | 113 |
| **Research and Remedies** ... ... ... ... | 114 |
| **Garden Cities for Consumptives** ... ... ... | 115 |
| A Plea for a National Scheme. By J. E. ESSLEMONT, M.B. | |
| **A Woman's Experience in a London General Hospital** ... ... ... ... ... ... | 118 |
| **York County Hospital** ... ... ... ... ... | 119 |
| The Committee's Reply to Sir Cooper Perry's Report. | |
| **The Effect of the Insurance Amendment Act on Hospital Finance** ... ... ... ... | 121 |
| **Gifts and Bequests** ... ... ... ... ... ... | 122 |
| **The Institutional Library** ... ... ... ... | 123 |
| **The Non-Panel Movement** ... ... ... ... | 124 |
| **Personalia** ... ... ... ... ... ... ... | 126 |
| **Obituary** ... ... ... ... ... ... ... | 126 |
| **The Institutional Worker** ... ... (Suppl.) | 1 |
| **Our Bureau of Information** ... ... ,, | 2 |
| **Buildings Contemplated** ... ... ,, | 3 |
| **The Book Market** ... ... ... ,, | 3 |

